# Quality assessment in general practice: diagnosis and antibiotic treatment of acute respiratory tract infections

**DOI:** 10.1080/02813432.2018.1523996

**Published:** 2018-10-08

**Authors:** Laura Trolle Saust, Lars Bjerrum, Volkert Siersma, Magnus Arpi, Malene Plejdrup Hansen

**Affiliations:** a Department of Clinical Microbiology, Herlev and Gentofte Hospital, University of Copenhagen, Herlev, Denmark;; b Section of General Practice and Research Unit for General Practice, Department of Public Health, University of Copenhagen, Copenhagen, Denmark;; c Research Unit for General Practice, Department of Clinical Medicine, Aalborg University, Aalborg, Denmark;; d Audit Project Odense, Department of Public Health, University of Southern Denmark, Odense, Denmark

**Keywords:** Antibiotics, general practice, respiratory tract infections, quality indicator, quality assessment, diagnosis, primary care

## Abstract

**Objective**: To investigate areas in need of quality improvement within the diagnostic process and antibiotic treatment of acute respiratory tract infections (RTIs) in Danish general practice by using quality indicators (QIs).

**Design and setting:** During a 4-week period in winter 2017, a prospective registration of patients diagnosed with RTIs was conducted in general practice in two regions of Denmark.

**Subjects:** Throughout the registration period each patient with symptoms of an RTI was registered. Information about age, symptoms and findings, duration of symptoms, the use and result of clinical tests, allergy towards penicillin, referral to secondary care and the antibiotic given were recorded.

**Main outcome measures:** Values and acceptable ranges for QIs focusing on the diagnostic process, the decision to prescribe antibiotics and the choice of antibiotics for patients with RTIs.

**Results:** Regarding the diagnostic process nearly all QIs for patients diagnosed with acute pharyngotonsillitis and pneumonia fell within the acceptable range. Contrarily, the diagnostic QIs for patients with acute otitis media and acute rhinosinusitis were outside the acceptable range. All indicators designed to measure overuse of antibiotics were outside the acceptable range and nearly all indicators assessing if patients were sufficiently treated fell within the acceptable range. QIs assessing use of the recommended type of antibiotic were only within the acceptable range for patients diagnosed with acute pharyngotonsillitis.

**Conclusion:** The findings indicate an overuse of antibiotics for RTIs in Danish general practice. Especially management of acute rhinosinusitis and acute bronchitis should be targeted in future quality improvement projects.

**KEY POINTS:**
- To improve antibiotic prescribing in general practice it is important to focus on both the diagnostic process and the prescribing patterns.
- The findings indicate an overuse of antibiotics for acute respiratory tract infections in Danish general practice.
- Especially the diagnostic process and antibiotic prescribing patterns for acute rhinosinusitis and acute bronchitis could benefit from future quality improvement interventions.

## Introduction

Antimicrobial resistance (AMR) has reached alarming levels in many parts of the world and is considered to be one of the largest threats to global health [1]. Reducing the unnecessary use of antibiotics is essential when facing AMR [2,3]. General practice is responsible for the majority of antibiotics prescribed to humans [4]. Antibiotic prescriptions are often issued to patients with acute respiratory tract infections (RTIs) even though the effect is small, if any [5,6]. Despite increasing awareness of AMR, antibiotic prescribing remains high in general practice [4]. When fighting the unnecessary use of antibiotics, insight into data about antibiotic prescribing in combination with physician education has proven to be effective [7]. Often information about the antibiotic prescribing patterns is provided as overall primary care antibiotic consumption. Few European countries are able to link antibiotic prescriptions to specific diagnoses [8,9]. Thus, a recent Danish study was able to characterise antibiotic prescribing patterns for RTIs in Danish general practice by linking prescription data with specific clinical indications [10]. However, the available data may not capture a sufficiently detailed picture of antibiotic prescribing. To improve the use of antibiotics it is essential to include information about both the diagnostic process and the decision behind prescribing in order to identify focus areas for future interventions.

Recently, quality indicators (QIs) for patients presenting to general practice with symptoms of an RTI have been developed [11]. This set of indicators comprises both the diagnostic process, the decision to prescribe antibiotics and the type of antibiotic prescribed. QIs are measurable elements that are proven to be a significant stimulus in the improvement of antibiotic prescribing [12]. By means of this set of newly developed QIs, we aimed to investigate areas in need of quality improvement within the diagnostic process and antibiotic treatment of RTIs in Danish general practice.

## Methods

### Setting

This study is part of a larger quality improvement project from Audit Project Odense (APO) [13] with the overall aim of improving the management of patients presenting to general practice with symptoms of an RTI [14]. All general practices in the North Denmark Region and the Region of Southern Denmark were invited to participate in a prospective registration of patients diagnosed with an RTI. Throughout a 4-week registration period from January to February 2017 each patient consulting general practice with symptoms of an RTI was registered. The diagnosis of RTI was based on the assessment and diagnostics of the attending healthcare professional. Only patients who consulted the clinic for the first time for the current RTI were included.

### Subjects and analyses

For each patient the following information was recorded: age, symptoms and findings, the duration of symptoms, the use and result of point-of-care tests (rapid Streptococcus A antigen detection (Strep A) test and C-reactive protein rapid (CRP) test), the use of tympanometry or x-ray, and if the patient had any known allergy towards penicillin. At the end of the consultation the final diagnosis and management (+/- antibiotics, type of antibiotic or referral to secondary care) were recorded.

In 2017 a set of 50 evidence-based QIs for the diagnosis and antibiotic treatment of RTIs were developed by means of a RAND Appropriateness Method. Each indicator was developed with a standard (acceptable range) that embodied acceptability of the particular performance addressed by that indicator [11]. Of these 50, 39 QIs were applicable to the data collected in the current study. Consequently, we calculated values (in percentage) for 39 QIs and compared these values with the acceptable ranges. 95% confidence intervals were calculated by transforming back to proportions the Normal approximation interval for the odds, so as to avoid intervals stretching below 0% or over 100%. Data were analysed using SAS 9.4.

## Results

A total of 44 (8.5%) of the invited practices participated in the study. During the registration period the participating 256 healthcare professionals registered 6124 consultations of patients presenting to general practice with symptoms of an RTI. Of these, 2323 patients were diagnosed with either acute pharyngotonsillitis, acute otitis media, acute rhinosinusitis, acute bronchitis, pneumonia or acute exacerbation of COPD, according to the second edition of International Classification of Primary Care (ICPC-2) [15]. The remaining patients with symptoms of an RTI were mainly diagnosed with common cold.

Table 1 shows the value and acceptable range for QIs focusing on the diagnostic process. Five out of the nine QIs were within the acceptable range. Nearly 90% of patients with acute pharyngotonsillitis fulfilling 2-3 modified Centor criteria (Temperature ≥38° C, tonsillar coating, tender cervical lymphadenitis, absence of cough, patient age (+ 1 point for age 3–14 years, -1 point for age less than 3 years, -1 point for age 45 years and above)) were examined with a strep A test. However, more than 70% of patients fulfilling 0-1 modified Centor criteria were also examined with a strep A test despite that Danish guidelines recommend not to test these patients. All four diagnostic QIs for patients with pneumonia fell within the acceptable range.

**Table 1.** Values and acceptable ranges for 9 quality indicators focusing on the diagnostic process in Danish general practice (2017).

Quality indicators^a^	Patients^a^ (n : n)	Value (95%CI) (%)	Acceptable range (%)
Patients with acute pharyngotonsillitis (*N* = 501)			
Patients fulfilling 2–3 modified Centor criteria^b^ examined with a Strep A test : patients fulfilling 2–3 modified Centor criteria^b^	214 : 241	88.8* (84.4–92.4)	80–100
Patients fulfilling 0–1 modified Centor criteria^b^ examined with a Strep A test : patients fulfilling 0–1 modified Centor criteria^b^	83 : 115	72.2 (63.6–79.8)	0–10
Patients with acute otitis media (*N* = 347)			
Patients >6 months^c^ with an evaluation of the eardrum mobility^d^: patients >6 months^c^	162 : 342	47.4 (42.1–52.7)	70–100
Patients with acute rhinosinusitis (*N* = 330)			
Patients with >10 days symptom duration or increasing symptoms after 5 days : patients	139 : 330	42.1 (36.9–47.5)	90–100
Patients with pneumonia (*N* = 530)			
Patients fulfilling <2 diagnostic criteria^e^: patients	39 : 530	7.4* (5.3–9.8)	0–20
Patients examined with a CRP test: patients	434 : 530	81.9* (78.5–85)	80–100
Patients with a CRP test <20 mg/l : patients	50 : 530	9.4* (7.1–12.1)	0–20
Patients examined with an X-ray of thorax : patients	38 : 530	7.2* (5.2–9.6)	0–30
Patients with acute exacerbation of chronic obstructive pulmonary disease (*N* = 147)			
Patients with acute exacerbation of dyspnea, coughing and/or expectoration greater than the daily variation : patients	113 : 147	76.9 (69.6–83.2)	90–100

* Within the acceptable range.

a Presented as numerator: denominator.

b Temperature ≥38 °C, tonsillar coating, tender cervical lymphadenitis, absence of cough, patient age (+1 point for age 3–14 years, -1 point for age less than 3 years, -1 point for age 45 years and above).

c Values are calculated for patients aged ≥1 year.

d Evaluated by tympanometry or pneumatic otoscopy.

e Symptoms of lower respiratory tract infection (cough +/− expectoration), emerging findings on examination of the chest (tachypnea, damping and/or auscultation of murmurs), signs of systemic disease (systemically unwell and/or temperature >38 °C).

Table 2 demonstrates the value and acceptable range for QIs focusing on the decision to prescribe antibiotics. Ten QIs were primarily designed to assess overuse of antibiotics (a low value is associated with a high quality of antibiotic prescribing). None of these indicators were within the acceptable range. For example, more than 60% of patients diagnosed with acute rhinosinusitis were prescribed an antibiotic and 37% of patients diagnosed with acute pharyngotonsillitis and fulfilling 0-1 modified Centor criteria were prescribed antibiotics, despite no antibiotic indication according to the Danish guidelines. Use of antibiotics for patients diagnosed with acute bronchitis also exceeded the 10% upper limit of acceptable use.

**Table 2.** Values and acceptable ranges for 16 quality indicators focusing on the decision to prescribe antibiotics in Danish general practice (2017).

Quality indicators^a^	Patients^a^ (*n : n*)	Value (95%CI) (%)	Acceptable range (%)
Patients with acute pharyngotonsillitis (*N* = 501)			
Patients with a positive Strep A test treated with antibiotics: patients with a positive Strep A test	247 : 248	99.6* (98.2–100)	90–100
Patients fulfilling 0–1 modified Centor criteria^b^ treated with antibiotics: patients fulfilling 0–1 modified Centor criteria^b^,^c^	42 : 115	36.5 (28.1–45.5)	0–10
Generally affected patients fulfilling 4–5 modified Centor criteria^b^ treated with antibiotics : generally affected patients fulfilling 4–5 modified Centor criteria^b^	30 : 31	96.8* (86.6–99.8)	90–100
Patients with acute otitis media (*N* = 347)			
Patients <6 months^d^ treated with antibiotics: patients <6 months^d^	3 : 4	75 (27.8–98.4)	90–100
Patients >6 months^e^ with no signs of fluid in the middle ear^6^ treated with antibiotics: patients >6 months^e^ with no signs of fluid in the middle ear^e^,^c^	1 : 3	33.3 (2.3–83.9)	0–10
Patients >6 months^e^ with ≤3 days of acute ear pain and no signs of fluid in the middle ear^f^ treated with antibiotics : patients >6 months^e^ with ≤3 days of acute ear pain and no signs of fluid in the middle ear^f^,^c^	0 : 0	–	0–10
Patients with acute rhinosinusitis (*N* = 330)			
Patients treated with antibiotics : patients^c^	213 : 330	64.5 (59.3–69.6)	5–10
Patients with a CRP test <10 mg/l treated with antibiotics: patients with a CRP test <10 mg/l^3^	34 : 87	39.1 (29.3–49.5)	0–10
Patients fulfilling <3 diagnostic criteria^g^ treated with antibiotics: patients fulfilling <3 diagnostic criteria^g^,^c^	108 : 197	54.8 (47.8–61.7)	0–10
Patients fulfilling >3 diagnostic criteria^g^ treated with antibiotics: patients fulfilling >3 diagnostic criteria^g^	105 : 133	78.9 (71.5–85.3)	80–100
Patients with <5 days symptom duration treated with antibiotics: patients with <5 days symptom duration^c^	47 : 80	58.8 (47.8–69.1)	0–10
Patients with acute bronchitis (*N* = 468)			
Patients treated with antibiotics: patients^c^	106 : 468	22.6 (19–26.6)	0–10
Patients with purulent expectorate treated with antibiotics: patients with purulent expectorate^c^	42 : 100	42 (32.6–51.8)	0–10
Patients with pneumonia (*N* = 530)			
Patients treated with antibiotics : patients	498 : 530	94* (91.7–95.8)	90–100
Patients <65 year fulfilling <2 diagnostic criteria^h^ treated with antibiotics: patients <65 year fulfilling <2 diagnostic criteria^h^,^c^	24 : 26	92.3 (78.1–98.7)	0–20
Patients with acute exacerbation of chronic obstructive pulmonary disease (*N* = 147)			
Patients fulfilling 2–3 Anthonisen criteria^i^ treated with antibiotics : patients fulfilling 2–3 Anthonisen criteria^i^	78 : 82	95.1* (89–98.5)	90–100

* Within the acceptable range.

a Presented as numerator: denominator;

b Temperature ≥38 °C, tonsillar coating, tender cervical lymphadenitis, absence of cough, patient age (+ 1 point for age 3–14 years, -1 point for age less than 3 years, -1 point for age 45 years and above).

c Quality indicator primarily designed to measure overuse of antibiotics.

d Values are calculated for children aged <1 year.

e Values are calculated for patients aged ≥1 year

f Evaluated by tympanometry or pneumatic otoscopy and the absence of ear discharge.

g Discolored nasal discharge and/or purulent secretion in the nasal cavities, strong localised pain, fever (>38 °C), elevated CRP, exacerbation after remission of the disease.

h Symptoms of lower respiratory tract infection (cough +/- expectorate), emerging findings on examination of the chest (tachypnea, damping and/or auscultation of murmurs), signs of systemic disease (systemically unwell and/or temperature >38 °C).

i Increased dyspnea, increased expectorate, increased purulence of expectorate. If only two criteria are met, one of them has increased purulence of expectorate.

Another six QIs focusing on the decision to prescribe antibiotics were primarily designed to assess if patients were treated with antibiotic when it is recommended (a high value is associated with a high quality of antibiotic prescribing). Four of these QIs were within the acceptable range and the remaining two fell very close to the acceptable range.

Value and acceptable range for QIs focusing on the choice of antibiotics are shown in Table 3. Two out of the 14 QIs were within the acceptable range. The proportion of patients treated with penicillin V (first-line antibiotic for most RTIs in Denmark) was calculated for five diagnoses, but was only within the acceptable range for patients diagnosed with acute pharyngotonsillitis. Prescribing penicillin V to patients with acute bronchitis and AOM deviated the most from the acceptable range by 34 and 30% respectively. A total of 19% of AOM patients treated with antibiotics were prescribed amoxicillin +/- clavulanic acid (within the acceptable range). We found that more than 75% of patients diagnosed with any of the RTIs and treated with macrolides had no known allergy to penicillin.

**Table 3.** Values and acceptable ranges for 14 quality indicators focusing on the choice of antibiotics in Danish general practice (2017).

Quality indicators^a^	Patients^a^ (*n : n*)	Value (95%CI) (%)	Acceptable range (%)
Patients with acute pharyngotonsillitis (*N* = 501)			
Patients treated with penicillin V: patients treated with antibiotics	326 : 345	94.5* (91.8–96.6)	90–100
Patients without known penicillin allergy treated with macrolides: patients treated with macrolides	4 : 10	40 (14.6–70)	0–10
Patients with acute otitis media (*N* = 347)			
Patients treated with penicillin V: patients treated with antibiotics	155 : 260	59.6 (53.6–65.5)	90–100
Patients treated with amoxicillin +/- clavulanic acid: patients treated with antibiotics	50: 260	19.2* (14.8–24.3)	0–20
Patients without known penicillin allergy treated with macrolides: patients treated with macrolides	5 : 6	83.3 (44.6–99)	0–10
Patients with acute rhinosinusitis (*N* = 330)			
Patients treated with penicillin V: patients treated with antibiotics	165 : 213	77.5 (71.5–82.7)	90–100
Patients without known penicillin allergy treated with macrolides: patients treated with macrolides	9 : 18	50 (28.1–71.9)	0–10
Patients with acute bronchitis (*N* = 468)			
Patients treated with penicillin V: patients treated with antibiotics	59 : 106	55.7 (46.2–65)	90–100
Patients treated with amoxicillin +/- clavulanic acid : patients treated with antibiotics	24 : 106	22.6 (15.4–31.2)	0–10
Patients without known penicillin allergy treated with macrolides: patients treated with macrolides	17 : 22	77.3 (57.4–91.2)	0–10
Patients with pneumonia (*N* = 530)			
Patients treated with penicillin V: patients treated with antibiotics	313: 498	62.9 (58.5–67)	80–100
Patients without known penicillin allergy treated with macrolides : patients treated with macrolides	74 : 88	84.1 (75.5-90.7)	0–10^†^
Patients with acute exacerbation of chronic obstructive pulmonary disease (*N* = 147)			
Patients without known penicillin allergy treated with quinolones: patients treated with quinolones	3 : 5	60 (19.9–91.9)	0–10
Patients with acute respiratory tract infection (*N* = 2323)			
Patients without known penicillin allergy treated with macrolides: patients treated with macrolides	113 : 150	75.3 (68–81.8)	0–10

*Within the acceptable range.

^a^Presented as numerator: denominator.

† This standard cannot be used during a mycoplasma epidemic.

## Discussion

### Main findings

According to the diagnostic process nearly all QIs for patients with acute pharyngotonsillitis and pneumonia fell within the acceptable range. Contrary, the diagnostic QIs for patients with AOM and acute rhinosinusitis were outside the acceptable range. All indicators designed to measure overuse of antibiotics were also outside the acceptable range, while nearly all indicators assessing if patients were sufficiently treated with antibiotics fell within the acceptable range. QIs assessing the use of first-line antibiotic were only within the acceptable range for patients with acute pharyngotonsillitis (95% treated with penicillin V). Finally, we found that most of the patients treated with macrolides had no known allergy to penicillin.

### Strengths and limitations

To our knowledge, this is the first study to investigate general practice´ management of patients with RTIs; comprising both the diagnostic process; the decision to prescribe and the type of antibiotic prescribed by means of QIs. Anyhow, this study also has some limitations.

The QIs used in this study were originally designed to describe the quality of GP´s management of patients with RTIs. However, in Danish general practice many patients with RTIs are consulted by other healthcare professionals, often nurses [14]. Consequently, we wanted to investigate the quality of diagnosis and antibiotic treatment of acute RTIs in general practice, regardless of the type of healthcare professional consulting the patients. As the same guidelines apply to all healthcare professionals working in general practice, we argue that it is acceptable to apply these indicators to data collected during an APO audit involving both GPs and other healthcare professions.

The healthcare professionals volunteering in this study may have been more interested in the topic being investigated and may be more likely to follow guidelines than non-participating healthcare professionals [16]. Consequently, the study participants are probably not representative of the average Danish healthcare professionals working in general practice, which could lead to an underestimation of the inappropriateness of antibiotic prescribing. Taking this into account the amount of inappropriate antibiotic prescribing exposed in this study may be even more extensive in general.

Ambivalent information on antibiotic prescribing was given in 27 (out of 2323) cases; we choose to view these as if antibiotics were not prescribed. This information bias could lead to the value of the QIs deviating further from the acceptable ranges. However, since the QIs designed to assess overuse of antibiotics all deviated from the acceptable ranges it is unlikely to bias our conclusions.

### Comparison with other studies

#### Quality indicators focusing on the diagnostic process

Nearly 90% of patients with acute pharyngotonsillitis fulfilling 2-3 modified Centor criteria were examined with a strepA test, indicating that strepA test is used in the case of moderate risk of infection with group A beta-hemolytic streptococci (GABHS) as recommended in Danish guidelines [17]. These guidelines also stress that the StrepA test should not be used when the risk of GABHS is very low (0-1 modified Centor criteria fulfilled). The fact that more than 70% of the patients fulfilling only 0-1 modified Centor criteria also were examined with a strepA test implies that strepA tests are used extensively, and not always in accordance with the Danish guidelines [17]. When patients with symptoms of a viral infection are examined with a strepA test there is a risk of a positive test (due to detection of commensal strains), and this group of patients will not likely benefit from antibiotic treatment [18]. As a positive strepA test often results in an antibiotic prescription it ultimately leads to antibiotic overuse. In Sweden, extensive use of strepA tests has also been observed and its possible influence on antibiotic overuse is being investigated [19].

More than 50% of all patients (aged > one year) diagnosed with AOM never had an evaluation of their eardrum mobility. Studies have shown that tympanometry or pneumatic otoscopy can improve the diagnostic quality of AOM [20]. However, Danish guidelines on management of children with AOM are conflicting. One guideline recommends that tympanometry or pneumatic otoscopy must be used as part of the diagnosis of AOM [21]. Contrary, another guideline states that GPs can consider using tympanometry or pneumatic otoscopy as part of the diagnosis [17]. It is possible that our results reflect the conflicting recommendations.

According to European Position Paper on Rhinosinusitis and Nasal Polyps [22] acute rhinosinusitis is subdivided into common cold, in which the duration of symptoms is less than ten days, and acute post-viral rhinosinusitis defined by deterioration of symptoms after five days or persistence beyond ten days. We found that nearly 60% of the patients diagnosed with acute rhinosinusitis have had symptoms for less than ten days nor any deterioration of symptoms, indicating that a significant amount of these patients were more likely to have been diagnosed with a common cold. These findings are in line with previous literature demonstrating that the diagnosis acute rhinosinusitis is used too often in general practice [23].

One has to keep in mind that the accurate diagnosis of pneumonia in general practice is challenging. Consequently there is a hypothesis of diagnostic labeling (misclassification) between acute bronchitis and pneumonia; that the physician tends to justify a prescription for acute bronchitis by labeling it pneumonia [24]. However, our findings contradict this hypothesis by indicating a high diagnostic quality of pneumonia. Few patients fulfilled less than two diagnostic criteria (symptoms of lower respiratory tract infection (cough +/- expectoration), emerging findings on examination of the chest (tachypnea, damping and/or auscultation of murmurs), signs of systemic disease (systemically unwell and/or temperature >38° C)) and had a CRP test <20 mg/l (Table 1). Furthermore, another study by Van Vugt et al found that a combination of physical examination with a CRP value improved the prediction of radiographic verified pneumonia [25].

#### Quality indicators focusing on the decision to prescribe antibiotics

All six QIs designed to assess if patients were sufficiently treated with antibiotics were very close to or within the acceptable range. In contrast, none of the QIs designed to assess overuse of antibiotics reached the acceptable range.

Nearly all patients with a positive strepA test were treated with antibiotics. Similarly, patients with a high risk of GABHS (patients fulfilling 4-5 modified Centor criteria) were sufficiently treated with antibiotics. However, 37% of patients with no indication for antibiotic treatment (patients fulfilling 0-1 modified Centor criteria) were also treated. Importantly, it has previously been demonstrated that Swedish GPs do not recall the Centor criteria [26] and our findings indicate that this might also be the issue in Danish general practice.

We found that 23% of patients diagnosed with acute bronchitis were treated with antibiotics. This is the lowest prescribing percentage of the six diagnoses studied. However, keeping in mind the lack of evidence to support the use of antibiotics for acute bronchitis [27] this is a rather high prescribing percentage.

A very small proportion (0.5-2%) of patients with acute rhinosinusitis develop acute bacterial rhinosinusitis (ABRS) and only when ≥3 specific diagnostic criteria are present the risk of ABRS is high [22]. We found that more than half of the patients fulfilling <3 of these criteria were treated with antibiotics. Our findings suggest that antibiotic prescribing in patients with acute rhinosinusitis is often not in agreement with guidelines and a considerable amount of these patients are probably exposed to overprescribing. Other European studies have shown similar results [28].

#### Quality indicators focusing on the choice of antibiotics

A decrease in penicillin V and an increase in amoxicillin are reported in the Danish Integrated Antimicrobial Resistance Monitoring and Research Program (DANMAP) report 2016 [29]. Importantly, the authors of the DANMAP report claim that if these trends are to continue amoxicillin will be the most commonly used antibiotic in Denmark within the next few years. We found that the use of penicillin V was within the acceptable range only for patients diagnosed with acute pharyngotonsillitis (95%) and deviated the most for patients with acute bronchitis and AOM. These findings are in line with a recent Danish registry-based study demonstrating a high use of penicillin V for acute pharyngotonsillitis (86%) and the lowest penicillin V use for acute bronchitis [10]. The relatively low use of penicillin V is not in accordance with Danish guidelines recommendations indicating that current choice of antibiotics could still be improved.

In Denmark, macrolide treatment is recommended only when patients have allergy to penicillin or in case of penicillin-resistant bacteria, sensible to macrolides, detected by microbiological testing. We found that more than 75% of the patients with acute RTIs treated with macrolides had no known penicillin allergy. It does not seem plausible to ascribe all of these 75% to cases of penicillin-resistant bacteria, indicating an overuse of macrolides. Of note, a Mycoplasma pneumoniae epidemic was observed in Denmark during winter 2016–2017, which may account for some of these prescriptions.

#### Antibiotic overprescribing in general practice

A Dutch study analysing GPs accordance with guidelines for patients with AOM, demonstrated both under- and overprescribing of antibiotics [30].In this study we found hardly any underprescribing and mainly overprescribing of antibiotics for RTIs. For example, many patients were prescribed antibiotics despite fulfilling only one criterion (diagnostic criteria or modified Centor criteria); although two or three criteria are required according to guidelines. It has previously been demonstrated that criteria for antibiotic treatment stated in guidelines are recognised by GPs as important items but not applied in a correct manner [30]. It may be difficult for the GPs to understand the value of combinations of multiple signs and symptoms.

Multiple possible reasons for overprescribing exists, one often mentioned is that clinicians feel pressured to prescribe antibiotics because of patients’ expectations [31]. The pressure to prescribe antibiotics can be real or perceived as a result of the clinician’s perception [31,32]. It has also been reported that clinicians tend to take a **‘**just in case**’** approach, meaning that they rather prescribe than not prescribe when they are uncertain about the prescribing decision [32]. Furthermore, studies have shown that many clinicians do not believe that antibiotic prescribing in primary care is responsible for the increasing problems with antibiotic resistance [33] and consequently not necessarily comply with new recommendations and national action plans.

### Perspectives

The findings of the study indicate an antibiotic overuse for patients presenting to Danish general practice with symptoms of an RTI. The QIs applied in this study represent a useful tool to evaluate and compare the quality of diagnosis and antibiotic treatment of RTIs in general practice. These QIs could provide national direction for improvement strategies such as public campaigns, physician education or organisational changes in healthcare. The Danish national action plan for antibiotics to humans issued in 2017 [34] identifies two main goals; to increase the proportion of penicillin V of the total antibiotic consumption and to reduce the number of redeemed prescriptions in general practice. Structural changes to an already existing data capture module for antibiotic prescriptions (Ordiprax) has been proposed as an initiative to reach the goals from the National action plan [34]. A systematic implementation of the QIs used in the current study into this module could meet the proposed initiatives. Furthermore, such implementation could become beneficial for future patients by assisting individual clinics striving to improve care as well as to monitor national surveillance targets on antibiotic use.
